# FAK Shutdown: Consequences on Epithelial Morphogenesis and Biomarker Expression Involving an Innovative Biomaterial for Tissue Regeneration

**DOI:** 10.3390/ijms22189774

**Published:** 2021-09-10

**Authors:** Xiaoling Wang, Thorsten Steinberg, Martin P. Dieterle, Imke Ramminger, Ayman Husari, Pascal Tomakidi

**Affiliations:** 1 Center for Dental Medicine, Division of Oral Biotechnology, Medical Center—University of Freiburg, Faculty of Medicine, University of Freiburg, Hugstetterstr. 55, 79106 Freiburg, Germany; xiaoling.wang@uniklinik-freiburg.de (X.W.); martin.dieterle@uniklinik-freiburg.de (M.P.D.); imke.ramminger@uniklinik-freiburg.de (I.R.); pascal.tomakidi@uniklinik-freiburg.de (P.T.); 2 Faculty of Biology, University of Freiburg, Schaenzlestr. 1, 79104 Freiburg, Germany; 3 Center for Dental Medicine, Department of Orthodontics, Medical Center—University of Freiburg, Faculty of Medicine, University of Freiburg, Hugstetterstr. 55, 79106 Freiburg, Germany; ayman.husari@uniklinik-freiburg.de

**Keywords:** epithelial morphogenesis, epithelial hyperplasia, proliferation, differentiation, focal adhesion kinase (FAK), siRNA, keratins, involucrin, filaggrin, biomaterial

## Abstract

By employing an innovative biohybrid membrane, the present study aimed at elucidating the mechanistic role of the focal adhesion kinase (FAK) in epithelial morphogenesis in vitro over 4, 7, and 10 days. The consequences of siRNA-mediated FAK knockdown on epithelial morphogenesis were monitored by quantifying cell layers and detecting the expression of biomarkers of epithelial differentiation and homeostasis. Histologic examination of FAK-depleted samples showed a significant increase in cell layers resembling epithelial hyperplasia. Semiquantitative fluorescence imaging (SQFI) revealed tissue homeostatic disturbances by significantly increased involucrin expression over time, persistence of yes-associated protein (YAP) and an increase of keratin (K) 1 at day 4. The dysbalanced involucrin pattern was underscored by ROCK-II^Ser1366^ activity at day 7 and 10. SQFI data were confirmed by quantitative PCR and Western blot analysis, thereby corroborating the FAK shutdown-related expression changes. The artificial FAK shutdown was also associated with a significantly higher expression of filaggrin at day 10, sustained keratinocyte proliferation, and the dysregulated expression of K19 and vimentin. These siRNA-induced consequences indicate the mechanistic role of FAK in epithelial morphogenesis by simultaneously considering prospective biomaterial-based epithelial regenerative approaches.

## 1. Introduction

Focal adhesion kinase (FAK), a cytoplasmic non-receptor tyrosine kinase, was independently discovered in 1992 by Steve Hanks, Jun-Lin Guan and Michael Schaller, as a highly tyrosine-phosphorylated substrate of the viral tyrosine protein kinase sarcoma (Src). In the field of mechanotransduction, i.e., the conversion of extracellular mechanical stimuli into intracellular biochemical signals, FAK is essential for the formation, maturation, and disassembly of integrin-based focal adhesions (FAs). The FAK-related FAs turnover mediates biological processes such as cell adhesion and motility. In addition, FAK is involved in further cell behavioral features, including apico-basal epithelial polarity, cell growth, proliferation, differentiation, and apoptosis [1,2,3,4]. In the context of carcinogenesis, FAK is overexpressed and highly active in many histological tumor samples as well as cancer cell lines [5]. It stimulates the downregulation of the epithelial cell adhesion molecule E-Cadherin, whose loss is a cornerstone in epithelial-mesenchymal transition (EMT) [6,7]. In addition, FAK supports the migratory phenotype of epithelial cells, thereby contributing to the migration and invasion of cancer cells as well as metastasis, as shown for epidermoid carcinoma and various squamous cell carcinomas [6,8,9,10,11]. Therefore, low molecular weight inhibitors of FAK activity, which prevent, e.g., its auto-phosphorylation at tyrosine residue 397 (Y397), are of high clinical interest [5]. 

The role of FAK has been intensively studied in various tissues, including skin. Generally, FAK knockout in mice is embryonically lethal due to mesodermal defects. Fibroblast-specific FAK knockout or FAK inhibition leads to less fibrosis and scarless wound healing. Consequently, increased expression of FAK in dermal fibroblasts is associated with fibrosis, hypertrophic scars, and inflammation during wound healing [12,13,14,15]. The conditional knockout of FAK in mouse-skin keratinocytes does not lead to any defects in wound healing. However, these keratinocytes cannot be grown in cell cultures since they are unable to adhere to the culture dish. In vivo, FAK-KO mice exhibit a specific phenotype, which is characterized by sebaceous gland hypoplasia, hair-cycle defects, a thin epidermis, and antitumorigenic properties [16,17,18,19,20]. Overall, epithelial morphogenesis is, however, not affected. Genetic analysis of these cells nonetheless revealed tremendous effects of FAK expression on epithelial function and integrity as exemplified by the modulation or neo-expression of keratins (e.g., Keratin 6), as well as changes in gene expression of matrix-remodeling genes (e.g., Matrix Metalloproteinases, Collagen 4a1) [14,21,22,23]. FAK overexpression in skin keratinocytes increases cellular spreading on various substrates [24,25]. 

Like skin, oral mucosal tissues also consist of keratinocytes and fibroblasts, although the morphology and biochemical properties such as the protein expression patterns of, e.g., skin and gingival epithelia vary considerably [26,27,28,29]. While, for instance, the epithelium of the hard palate is keratinized, the buccal mucosal epithelium is nonkeratinized and lacks a stratum granulosum and a stratum corneum. Comparable to skin, the hard palate is ortho-keratinized, i.e., is devoid of cell nuclei within the stratum corneum [30]. Contrary to that, the gingiva exhibits a para-keratosis, which is characterized by cell nucleus-harboring keratinocytes within the stratum corneum [31]. Of note, oral epithelial morphogenesis and regeneration is a lifelong process and is maintained by the coordinated, homeostatic balance of keratinocyte proliferation and differentiation [32]. 

The gingiva normally harbors 20–30 keratinocyte cell layers and has a faster cell turnover than skin [33,34]. Importantly, data on the biological role of FAK in the gingiva is scarce. Wound closure works very fast and without scar formation, which might be related to FAK function in gingival fibroblasts (GFs) [35]. In oral squamous cell carcinomas, which derive from keratinocytes within the oral cavity, FAK promotes cell survival, proliferation, cell adhesion, invasion, and metastasis. FAK is also important for the interaction with the tumor microenvironment and its overexpression is associated with resistance to radio- and chemotherapy, as well as local recurrence [11,36,37]. Apart from the cancer context, gingival epithelial morphogenesis in the context of FAK function as well as the impact of FAK depletion on keratinocyte protein expression patterns has, to the best of our knowledge, not been studied so far.

In stratified epithelia, such as the epidermis and gingiva, differentiation is accompanied by morphological changes, which occur during vertical keratinocyte migration from the basal cell layer up to the uppermost cell sheet, and are reflected by a progressive cell flattening [32,38]. In conjunction with the morphological alterations, differentiation is coupled to the synthesis of biomolecules, among which the intermediate filament (IF) proteins keratin (K) 1 and K10 discriminate early from late stage and terminally differentiated keratinocytes [39,40]. The latter are best characterized by involucrin and filaggrin expression [41]. Involucrin is a soluble protein precursor of the cornified envelope in differentiating keratinocytes and can be detected histochemically from the early spinous layer on [42]. Filaggrin is involved in the maintenance of the epithelial barrier function and aggregates keratin filaments, thereby contributing to the structural and mechanical integrity of the stratum corneum [43]. As mentioned above, keratins fulfil important mechanical functions and are responsible for the keratinocyte’s resistance to shear and tensile forces [44]. 

Within the epithelia of the oral mucosa, keratin 19 (K19), normally found in simple epithelia such as the respiratory epithelium, is expressed in basal and suprabasal cells of the primary junctional epithelium and in basal cells of the sulcular epithelium [45]. K19 is also involved in the induction of epithelial-mesenchymal transition (EMT) [46]. Epithelial cells undergoing EMT additionally express vimentin, which normally represent the IFs of mesenchymal cells, e.g., connective tissue fibroblasts [47,48]. Similar to FAK, vimentin has also been shown to participate in cell migration and in the stabilization of focal contacts (FAs) [49,50]. Due to the above-mentioned interrelationship of FAK knockout and IF expression and function in keratinocytes, it is therefore of high interest to elucidate the role of FAK in oral epithelial morphogenesis.

In addition to growth factors like epidermal growth factor (EGF) [51,52], keratinocyte proliferation, as experimentally detectable by markers such as serine 10-phosphorylated histone 3(pHH3^Ser10^), can be stimulated by the mechanoresponsive transcriptional co-activator yes-associated protein 1 (YAP1, herein YAP) [53,54]. YAP, in conjunction with its cellular homologue transcriptional co-activator with PDZ motif (TAZ), has initially been identified as a member of the canonical HIPPO pathway in the fruit fly *Drosophila melanogaster*. In mammals, the YAP-regulatory kinases Lats1/2 and Mst1/2 trap YAP in the cytoplasm through phosphorylation and favor its interaction with the 14-3-3 σ-protein or induce its ubiquitin-mediated proteasomal degradation. In addition, cytoplasmic YAP acts as a regulator of the proliferative function of β-catenin. Apart from changes in its phosphorylation status, YAP/TAZ can also be regulated by biophysical stimuli, i.e., mechanical signals from the cell’s environment or intracellular cytoskeletal tension [55,56]. In this context, several recently published studies have underscored the tight regulatory interdependency of FAK and YAP, rendering YAP an important indicator of FAK function and vice versa [57,58]. 

The transcription of YAP-target genes, which comprise cellular key players like Cyclin D1 and c-myc, is only possibly, if YAP/TAZ enters the nucleus. It could be demonstrated that YAP nuclear presence was directly coupled to the activity of ROCK-II and that transcriptional activity depends on Rho-associated protein kinase I (ROCK-I) [59]. This suggests that YAP function is regulated by both of these actin cytoskeleton regulating Rho-GTPase effectors, which are also downstream targets of FA-related integrin-FAK signaling [60]. As mentioned above, in vitro cell culture studies revealed that the stretch-induced phosphorylation (*p*) of FAK at its autophosphorylation site tyrosine 397 (Y397) depends on the RhoA/ROCK signaling pathway [61]. Regarding keratinocyte differentiation, inhibition of ROCK-II activity resulted in a loss of terminal differentiation [62]. 

Due to its, so far, incompletely understood function in the gingival epithelium, its enormous impact on cell behavior and gene expression, as well as its interaction with HIPPO signaling and the cytoskeletal regulators ROCK-I and ROCK-II, FAK is as an optimal candidate to analyze in the context of oral epithelial morphogenesis and tissue regeneration. 

In the past, cocultures of GFs and gingival keratinocytes (GKs) proved to be valuable tools to establish organotypic epithelial equivalents of the gingival mucosa, which showed protein expression patterns comparable to the native mucosa [26,27,33,63,64,65]. Gingival epithelial morphogenesis, differentiation, as well as integrity are in this regard highly dependent on the presence of the fibroblasts. Three-dimensional gingival models with de-epidermized dermis, fibroblast-collagen-matrixes, or synthetic biomaterials are described in the literature [66,67]. The latter are especially advantageous, since tailored and reproducible fabrication of the biomaterial allows for site-specific adaptation of biochemical and biophysical properties of the material and omits the need for donor tissue [68]. With respect to a suitable biomaterial supporting the formation of in vitro epithelial equivalents, we could show in previous studies that electrospun nanofiber-based gelatin-nonwoven mats promoted oral gingival epithelial formation both in vitro and in vivo [32,69]. 

The present proof-of-principle study focuses on the mechanistic role of FAK in gingival epithelial morphogenesis and employs our innovative gelatin-nonwoven biomaterial as a substrate for in vitro epithelial tissue formation based on a GKs/GFs coculture. The histological and molecular consequences of siRNA-mediated FAK depletion show for the first time, how FAK is involved in the regulation of gingival epithelial proliferation and differentiation, and broaden the mechanistic insights into FAK function within epithelia. The data are an important basis for further analysis of the tissue-specific characteristics of FAK function and are an indispensable precondition for future biomaterial-based strategies in epithelial regenerative medicine.

## 2. Results

### 2.1. siRNA-Mediated FAK Shutdown 

The non-receptor tyrosine kinase FAK exerts diverse functions in cellular physiology and pathophysiology ranging from cell adhesion and differentiation to proliferation and carcinogenesis. To investigate its role in oral gingival epithelial development and biomarker expression, FAK availability was downregulated via application of a FAK-specific siRNA to the cells for different periods of time. 

siRNA-mediated FAK knockdown experiments were performed via a transfection-based approach, leading to a post-transcriptional gene silencing of the FAK mRNA transcripts. Thus, RNA interference leads to an overall reduction of FAK protein and inhibits all FAK functions. The optimal inhibitory concentration of the siRNA was determined to be 25 nM with the help of preliminary experiments in GK monolayer cultures (supplementary data, Figure S1). The rest of the below-described biomarkers were not analyzed in monolayer GK culture, since their expression is dependent on the respective epithelial cell layer and should therefore be analyzed in 3D samples.

**Figure 1:**
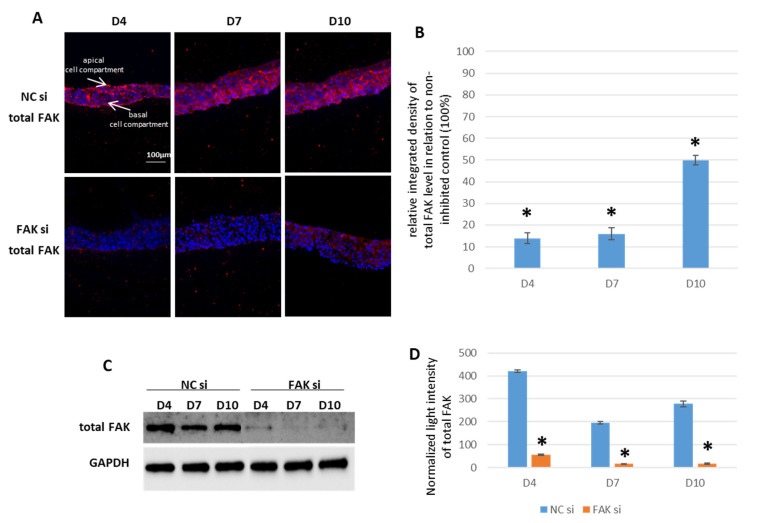
siRNA-mediated inhibition of FAK in GK-based cocultures. Cells were transfected with FAK-siRNA and incubated for 48 h before usage in the coculture setup for further 4, 7, or 10 days. Shown in (**A**) are representative IIF images of cryofixed sections of FAK-siRNA-treated versus nontreated cocultures stained with an anti-FAK antibody after the indicated periods of time (the orientation of the epithelia is the same in all figures). The bar graph in (**B**) shows the SQFI of the data from (**A**) by measuring the relative integrated fluorescence density of the total FAK fluorescence signal and compares the FAK-siRNA-inhibited coculture with the noninhibited control (set to 100%). Shown in (**C**) is a representative Western blot from whole cell keratinocyte lysates depicting total FAK in FAK-siRNA-inhibited cocultures and the noninhibited control for the indicated growth periods together with the loading control GAPDH. The bar graph in (**D**) illustrates the amount of total FAK by measuring the normalized light intensity of the respective bands. (*n* = 3 for all experiments, values are depicted as mean ± SEM; * = *p* < 0.05. Scale bars represent 100 µm. NC = negative control; D = day).

Subsequently, epithelial equivalents as cocultures of GKs and GFs were cultivated on nonwoven biohybrid membranes as described in the Section 4. siRNA treatment with 25 nM also led to an efficient FAK shutdown in epithelial equivalents at all points of time under study. Figure 1 shows a significant cytoplasmic depletion of total FAK protein in semiquantitative fluorescence imaging (SQFI) and a reduction in whole cell lysates as detected by Western blot (WB) experiments (Figure 1A,B: SQFI and Figure 1C,D: WB). 

Next, we tested for the effects of a low molecular weight inhibitor of FAK on gingival keratinocytes and epithelial equivalents. As it is known from the literature that pharmacological inhibition can lead to different biological effects on cells and tissues when compared to siRNA-based experimental approaches, we wanted to elucidate if this is also true for FAK inhibition [58]. Contrary to the siRNA, the FAK inhibitor Y15 only prevents FAK’s autophosphorylation at Y397, which is important for some but not all FAK functions [70]. Y15 pretesting in GK monolayer cultures revealed no cytotoxic effects but residual pFAK Y397 protein levels at 10 µM Y15 with a gradual increase in FAK Y397 abundance at 48 h after inhibitor application. However, pFAK Y397 was almost absent at 20 µM of Y15 inhibitor (supplementary data, Figure S2A). Treatment of the epithelial equivalent GKs with 10 µM Y15 yielded intact epithelia, while 20 µM of Y15 resulted in unsatisfactory phenotypes and histological architectures (supplementary data, Figure S2C). Surprisingly, the epithelia of Y15-treated cells exhibited increased levels of pFAK Y397 protein, particularly at day 4 (supplementary data, Figure S2B). This paradoxical finding may be explained by a compensating paracrine signaling of the fibroblasts in the coculture, as demonstrated for fibroblast-derived EGF, which has been shown to target FAK Y397 [71,72]. Due to this uncertainty and the potentially deleterious effects of the Y15 inhibitor for the experimental results with epithelial equivalents, FAK inhibition via specific siRNA with the above-mentioned concentration was chosen for subsequent studies on FAK shutdown-related consequences on gingival epithelial cocultures. 

### 2.2. Effect of FAK-siRNA on Oral Epithelial Morphogenesis

**Figure 2:**
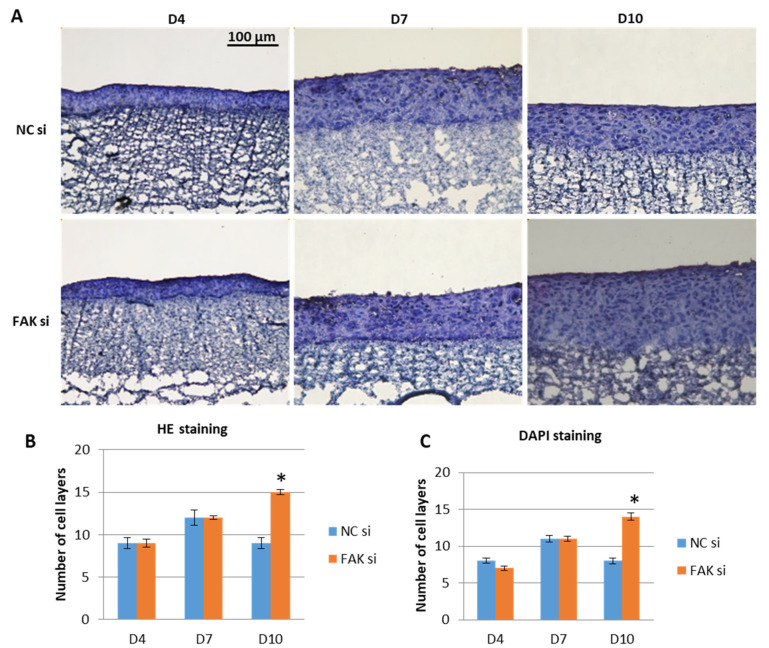
siRNA-mediated inhibition of FAK in GK-based cocultures. Cells were transfected with FAK-siRNA and incubated for 48 h before usage in the coculture setup for a further 4, 7, or 10 days. Shown in (**A**) are representative cryofixed and HE stained sections of FAK-siRNA-treated versus nontreated cocultures at the indicated points of time. The number of cell layers were counted manually in the HE sections, and the corresponding statistical analysis is shown in (**B**). DAPI stained sections were also evaluated for the number of cell layers and analyzed statistically as shown in (**C**). Bar graphs illustrate the increase in the number of cell layers over time and depict the statistically significant difference of FAK-siRNA-inhibited cocultures versus the noninhibited control at day 10. (*n* = 3 for all experiments, values are depicted as mean ± SEM; * = *p* < 0.05. Scale bars represent 100 µm. NC = negative control; D = day).

After having proven the general feasibility of the FAK knockdown within in vitro epithelial equivalents, the next step was to examine the effect of FAK-specific siRNA on oral epithelial morphogenesis. To this end, GK-derived epithelial equivalents were analyzed with regard to their number of cell layers by either HE or cell nuclei DAPI stain at days 4, 7 and 10. The cell layers were counted manually and averaged by recording cell nuclei in the apico-basal direction in three different sectors of each section (for details see Material and Methods). As exemplified in Figure 2 for HE staining, tissue morphogenesis in untreated controls showed an expected pattern as usually recognized in in vivo-like coculture models (own observations). The number of cell layers increased between day 4 und day 7 and remained constant at day 10.

In contrast, a steady increase in cell layers in FAK-siRNA-treated GK epithelia could be detected within the observed period (Figure 2A,B). In the interepithelial comparison, i.e., control versus siRNA-treated epithelia, quantification of the cell layers showed similar numbers on day 4 and 7 (Figure 2A,B). Of interest, FAK-siRNA administration yielded significantly higher numbers of epithelial cell layers on day 10 when compared to controls, regardless of the visualization method, i.e., HE or DAPI staining. In detail, while in FAK-siRNA-treated GKs up to 15 cell layers were counted in the case of HE staining (Figure 2B), and 14 cell layers through DAPI detection (Figure 2C), nontreated epithelial equivalent counterparts showed a maximum number of cell layers of 9 (HE staining; Figure 2A), and 8 (DAPI staining; Figure 2C). Both modes of quantification revealed an increase in the number of cell layers of approximately 40% in epithelial equivalents following the administration of FAK-siRNA to GKs. Phenotypically, the FAK-siRNA-treated samples at day 10 resembled hyperplastic tissue, indicating that FAK is involved in the regulation of cell proliferation and the control of epithelial thickness in gingival epithelia. On the cellular level, morphological differences between the siRNA-treated keratinocytes and the control samples could not be detected.

### 2.3. Consequences of FAK Knockdown on Differentiation Biomarkers

**Figure 3:**
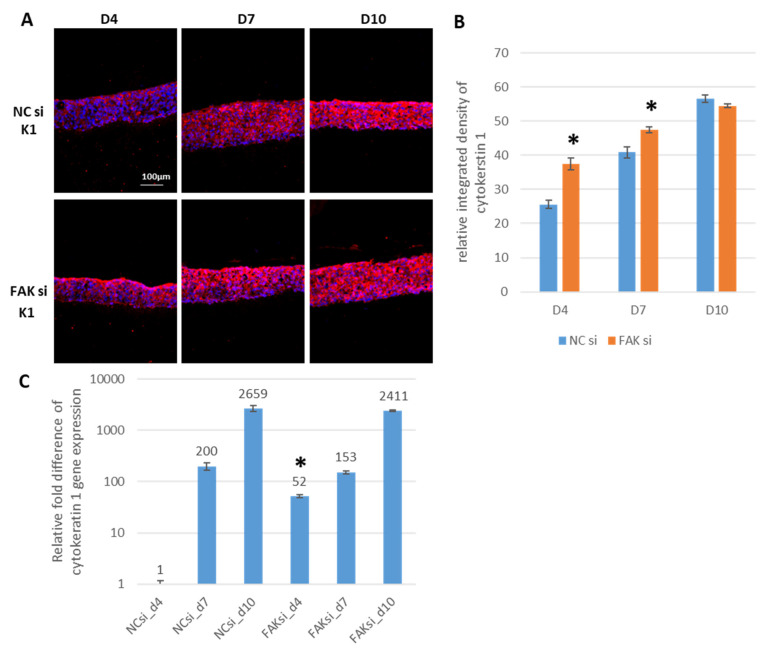
siRNA-mediated inhibition of FAK in GK-based cocultures and the influence on cytokeratin 1 (K1) expression and distribution. Cells were transfected with FAK-siRNA and incubated for 48 h before usage in the coculture setup for a further 4, 7, and 10 days. Shown in (**A**) are representative IIF images of cryofixed sections of FAK-siRNA-treated versus nontreated cocultures, stained with an anti-cytokeratin 1 antibody after the indicated periods of time. The bar graph in (**B**) shows the SQFI of the cytokeratin 1 fluorescence signals by measuring the relative integrated fluorescence density. FAK-siRNA inhibited cocultures are compared to the noninhibited control. Shown in (**C**) is the relative fold difference of gene expression of cytokeratin 1. Day 4 of the control specimens was used for normalization. (*n* = 3 for all experiments, values are depicted as mean ± SEM; * = *p* < 0.05. Scale bars represent 100 µm. NC = negative control; D = day).

As FAK knockdown led to an increase in cell layers, the next step was to gain an overview of the differentiation state of the epithelial equivalents. To investigate the consequences of FAK-directed RNA interference (RNAi) on the expression of typical keratinocyte biomarkers that indicate the various stages of epithelial differentiation, cytokeratin 1 (K1), as a representative of early GK differentiation, was detected by IIF (protein level, Figure 3A) and qPCR (transcriptional level, Figure 3C), respectively. Quantification of mainly cytoplasmic fluorescence signals via SQFI indicated almost equal K1 protein amounts in epithelial equivalents at day 10 in siRNA-treated samples versus controls (Figure 3B). 

Of interest, however, detectable K1 protein quantities could be discriminated at a significant level on day 7 and especially on day 4 (Figure 3B). This points into the direction that the loss of FAK regulatory activity goes along with a temporally advanced K1 expression. With respect to its spatial distribution, K1 showed an increase in abundance within suprabasal and apical epithelial cell layers already at day 4 in response to FAK-siRNA administration (Figure 3A: compare upper with lower panel, D4). The drastic difference detected for K1 on the protein level at day 4 was supported by qPCR results, which indicated a 52-fold increase in K1 gene transcription in the epithelia of FAK-siRNA-treated GKs (Figure 3C, FAK si_d4).

**Figure 4:**
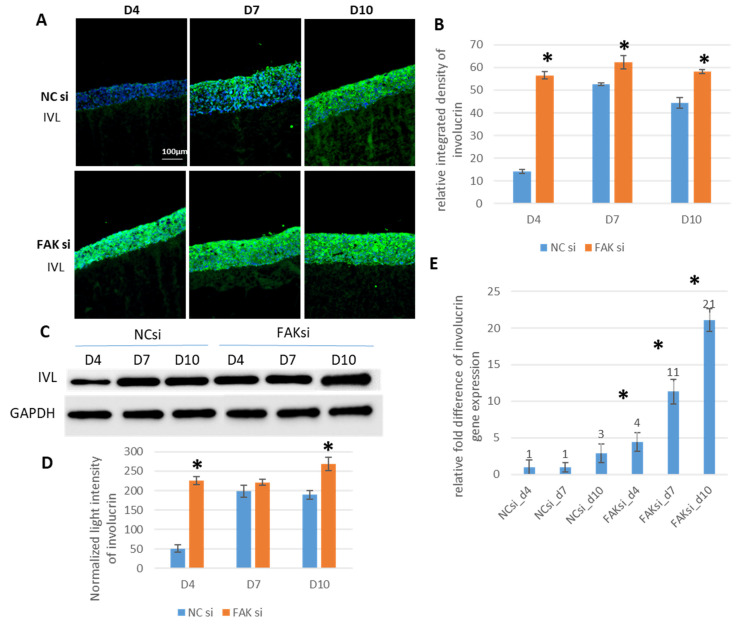
siRNA-mediated inhibition of FAK in GK-based cocultures and the influence on involucrin (IVL) expression and distribution. Cells were transfected with FAK-siRNA and incubated for 48 h before usage in the coculture setup for a further 4, 7, or 10 days. Shown in (**A**) are representative IIF images of cryofixed sections of FAK-siRNA-treated versus nontreated cocultures, stained with an anti-involucrin antibody after the indicated periods of time (4, 7, or 10 days). The bar graph in (**B**) shows the corresponding SQFI analysis by measuring the relative integrated fluorescence density of the involucrin fluorescence signal and compares the FAK-siRNA inhibited coculture with the noninhibited control. Shown in (**C**) is a representative Western blot of whole cell lysates from keratinocytes depicting the protein amount of involucrin in FAK-siRNA inhibited cocultures and the noninhibited controls together with the loading control GAPDH at the indicated points of time. The bar graph in (**D**) illustrates the involucrin Western blot quantification by measuring the normalized light intensity of the respective bands. (**E**) Represents the quantitative PCR analysis of the calculated mRNA-expression levels of involucrin as the relative fold difference when compared to the control samples at day 4. (*n* = 3 for all experiments, values are depicted as mean ± SEM; * = *p* < 0.05. Scale bars represent 100 µm. NC = negative control; D = day).

Next, involucrin protein levels and gene expression were tested, to analyze whether FAK-siRNA application affects terminal GK differentiation in the epithelial equivalents. Regarding the epithelia of FAK-siRNA-exposed GKs, the semi-quantification of cytoplasmic and membrane-associated involucrin protein, as detected by IIF (Figure 4B) and WB (Figure 4C), demonstrated significantly increased levels at almost each point of time when compared to controls. Involucrin detection by WB analysis showed higher protein amounts on day 4, 7, and day 10. These differences were statistically significant on day 4 and 10 (Figure 4C), as confirmed by WB quantification (Figure 4D). Additionally, FAK-directed RNAi resulted in an involucrin presence throughout almost the entire epithelium from day 4 onwards, whereas it was foremostly localized in apical cell layers in the control epithelia on day 4 and 7 (Figure 4A: compare upper panel without RNAi with lower panel FAK RNAi).

For quantitative PCR analysis, the relative involucrin RNA expression in the control samples was set to 1 at day 4. Relative to that, it remained constant at day 7 and increased by a factor of 2.9 at day 10 in the nontreated epithelial equivalents. When exposed to FAK-siRNA, treated epithelial equivalents showed a 4.4-fold increase in involucrin gene expression at day 4, an 11.3-fold increase at day 7, and a 21.1-fold increase at day 10 (Figure 4E). The determined RNA expression levels indicate a continuous, statistically significant higher involucrin transcription following application of FAK-siRNA. 

**Figure 5:**
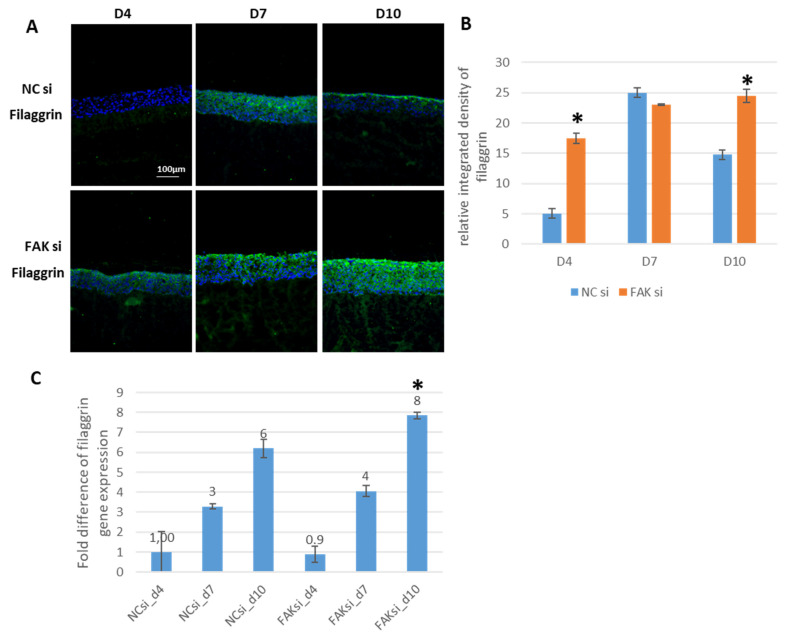
siRNA-mediated inhibition of FAK in GK-based cocultures and the influence on filaggrin (FLG) expression and distribution. Cells were transfected with FAK-siRNA and incubated for 48 h before usage in the coculture setup for a further 4, 7, or 10 days. Shown in (**A**) are representative IIF images of cryofixed sections of FAK-siRNA-treated versus nontreated cocultures, stained with an anti-filaggrin antibody after the indicated periods of time. The bar graph in (**B**) shows the SQFI by measuring the relative integrated fluorescence density of the filaggrin fluorescence signal and compares the FAK-siRNA inhibited cocultures with the noninhibited controls. In (**C**), the calculated mRNA-expression from the qPCR analysis is depicted as the relative fold difference of gene expression of filaggrin on day 4 of the control samples. (*n* = 3 for all experiments, values are depicted as mean ± SEM; * = *p* < 0.05. Scale bars represent 100 µm. NC = negative control; D = day).

Another well-established marker protein of terminally differentiated keratinocytes is filaggrin. To test whether cytoplasmic filaggrin shows a comparable biological behavior to involucrin under FAK inhibition, the aforementioned experiments were repeated with a focus on filaggrin. Interestingly, the SQFI analysis of histological sections revealed significantly higher levels of filaggrin protein at day 10 in siRNA-treated GK epithelial equivalents (Figure 5A: compare negative control (upper panel), versus FAK-directed RNAi (lower panel), and Figure 5B). Of note, filaggrin was restricted to the apical cell layer(s) in control cultures at day 10, while it showed strong fluorescence signals within the apical cell layers and an extended detectability into the basal region of the epithelial equivalents in the case of FAK-siRNA-treated samples (Figure 5A, D10). 

In addition, filaggrin mRNA gene expression supported the SQFI data and showed significant increased expression FAK-siRNA-treated samples at day 10 (Figure 5C).

Taken together, the findings on the differentiation markers K1, involucrin, and filaggrin show that FAK knockdown leads to remarkable shifts in the spatiotemporal expression of these proteins. Functionally, the observed patterns point in the direction that the depletion of FAK protein promotes an earlier differentiation of keratinocytes, which can be detected in more basal cell layers than under physiological conditions.

### 2.4. Impact of FAK-siRNA on Vimentin and K19

**Figure 6:**
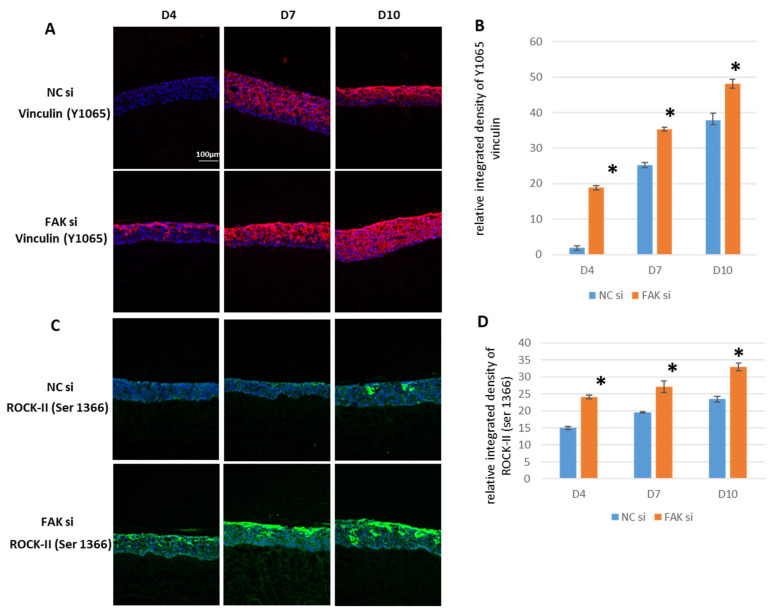
siRNA-mediated inhibition of FAK in GK-based cocultures and the influence on vimentin and cytokeratin 19 (K19) expression and distribution. Cells were transfected with FAK-siRNA and incubated for 48 h before usage in the coculture setup for a further 4, 7, or 10 days. Shown in (**A**) are representative IIF images of cryofixed sections of FAK-siRNA-treated versus nontreated cocultures, stained with an anti-vimentin antibody. In (**C**), epithelial equivalents are stained with an anti-cytokeratin 19 antibody after the indicated periods of time. The bar graph in (**B**) shows the SQFI by measuring the relative integrated fluorescence density of the vimentin fluorescence signal. Likewise, (**D**) represents the same analysis for the cytokeratin 19 fluorescence signal and compares the FAK-siRNA-inhibited cocultures with the noninhibited controls. (*n* = 3 for all experiments, values are depicted as mean ± SEM; * = *p* < 0.05. Scale bars represent 100 µm. NC = negative control; D = day).

Since the observed changes in keratinocyte differentiation and the tissue hyperplasia may be indicative of transformative cellular processes that might be relevant in the context of carcinogenesis, the mesenchymal IF vimentin and the keratin K19 were examined next. The experimental findings of cultured keratinocytes revealed an expression of vimentin, which is involved in EMT and cell migration, but also in FA stabilization [49,50]. Thus, we investigated whether FAK-directed RNAi has an impact on vimentin expression. Although vimentin expression and cytoplasmic localization appeared quite comparable between controls and treated samples at day 4, it persisted primarily in basal epithelial layers of epithelial equivalents of siRNA-treated GKs (Figure 6A: upper panel, NCsi and lower panel, FAKsi). Vimentin fluorescence detection by SQFI revealed that in the case of FAK RNAi, vimentin protein amounts were significantly higher at day 7 and day 10 when compared to controls (Figure 6B). 

Next, cytoplasmic K19 expression was analyzed, due to its putative association with FAK modulation [50]. SQFI-based IIF analysis (Figure 6C: upper panel, NCsi and lower panel, FAKsi) indicated a permanent and significantly higher epithelial K19 protein expression in response to FAK-directed RNAi (Figure 6D). Morphologically, K19 was detected primarily in apical epithelial layers.

The findings on vimentin and K19 again underscore the involvement of FAK in the regulation of keratinocyte differentiation during gingival epithelial morphogenesis.

### 2.5. Effect of FAK RNAi on Vinculin and Phosphorylated ROCK-II^Ser1366^

Since FAK knockdown had remarkable consequences on oral epithelial morphology and the differentiation of keratinocytes, as represented by K1, K19, involucrin, filaggrin, and vimentin expression patterns, we next wanted to shed light into the mechanistic basis of these observations. As FAK is a master regulator of cellular mechanotransduction, we hypothesized that signal transduction of mechanobiologically relevant signaling pathways could be responsible for the cell behavioral features of the siRNA-treated keratinocytes. 

**Figure 7:**
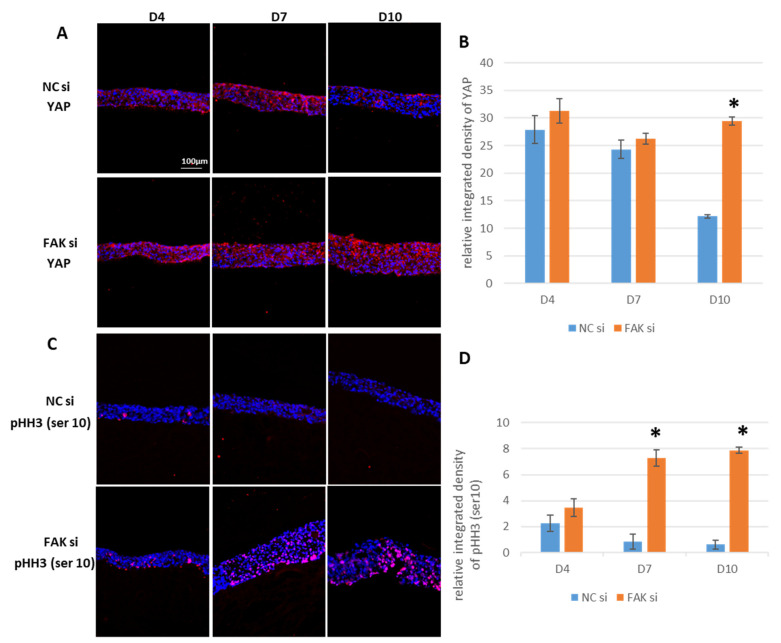
siRNA-mediated inhibition of FAK in GK-based cocultures and the influence on vinculin and ROCK-II expression and distribution. Cells were transfected with FAK-siRNA and incubated for 48 h before usage in the coculture setup for a further 4, 7, or 10 days. Shown in (**A**) are representative IIF images of cryofixed sections of FAK-siRNA-treated versus nontreated cocultures stained with an anti-vinculin Y1065 antibody after the indicated periods of time. Similarly, (**C**) depicts representative IIF images of cryofixed sections of FAK-siRNA-treated versus nontreated cocultures, stained with an anti-pROCK-II^Ser1366^ antibody after the indicated periods of time. The bar graph in (**B**) represents the SQFI by measuring the relative integrated fluorescence density of the vinculin fluorescence signal. Likewise, (**D**) shows the pROCK-II^Ser1366^ fluorescence signal and compares the FAK-siRNA inhibited coculture with the noninhibited control. (*n* = 3 for all experiments, values are depicted as mean ± SEM; * = *p* < 0.05. Scale bars represent 100 µm. NC = negative control; D = day).

Vinculin is a cytosolic protein both involved in FAs and adherens junctions (AJs). AJs are lateral cell-to-cell contacts, which are mandatory for keratinocyte differentiation in stratified epithelia and force transmission between cells [73]. As both FAK and vinculin are intimately linked to actin cytoskeleton regulation and thus cell morphology, spreading, and mechanotransduction, vinculin is an interesting protein to analyze in the context of FAK-knockdown-related changes. Phosphorylation at tyrosine (Y) residue 1065 (Y1065) of vinculin is an indicator of both force transmission and FA maturation. Therefore, the activation status of vinculin was checked by detecting Y1065 phosphorylation in the epithelial equivalents (Figure 7A,B). Activated vinculin was constantly increased at significant levels in the presence of FAK-siRNA as detected by SQFI. Here, particularly on day 4, a strong apical signal abundance of Y1065 fluorescence was detectable (Figure 7A, D4, FAKsi). In contrast to that, the control epithelial equivalents exhibited almost no detectable signal (Figure 7A, D4, Negative Control (NCsi)). 

Moreover, FAK phosphorylation and thus activity is strongly coupled to the cytoplasmic Rho/ROCK system as stated in the introduction section [61]. As mentioned, ROCK-I and ROCK-II activity was shown to regulate terminal keratinocyte differentiation and the subcellular localization and function of YAP. The latter in turn exerts regulatory activity on FAK. It was therefore of great interest to test the activity status of ROCK-II in response to FAK inhibition, to see whether this signaling axis responded to the intervention. To this end, phosphorylated (*p*) ROCK-II^Ser1366^ was tested (Figure 7C,D), because it directly indicates the kinase activity of ROCK-II. SQFI at day 4 already showed significantly higher protein quantities of phosphorylated ROCK-II in response to FAK RNAi (Figure 7C: compare D4, NCsi with D4, FAKsi). The histological appearance proved to be a predominantly apical localization of the protein of interest. Furthermore, significantly higher amounts of pROCK-II^Ser1366^ were also detected at day 7 and day 10, when compared to the control epithelial equivalents (Figure 7C: compare D7 and D10, NCsi with D7 and D10, FAKsi and Figure 7D). 

### 2.6. Maintenance of YAP and pHH3^Ser10^ in FAK-siRNA-Treated Epithelial Equivalents 

**Figure 8:**
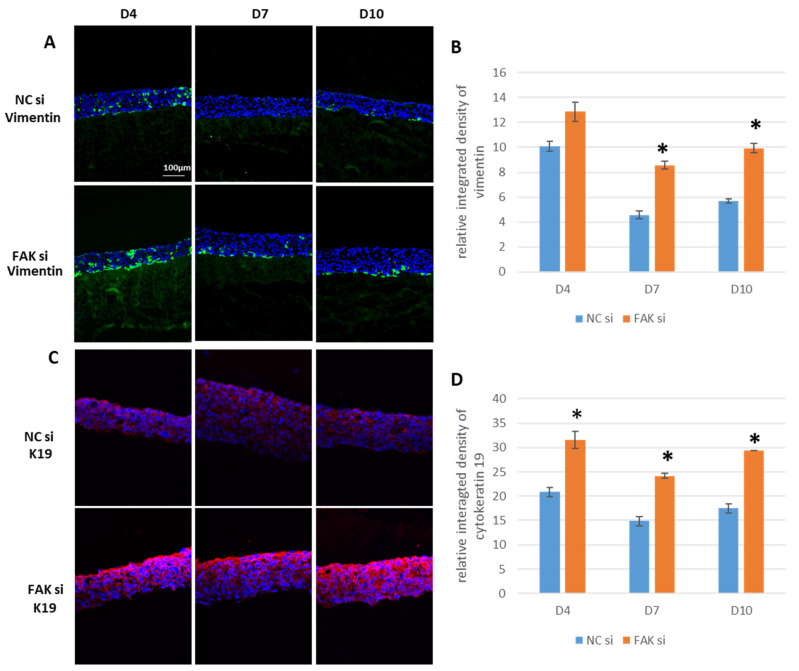
siRNA-mediated inhibition of FAK in GK-based cocultures and the influence on YAP and pHH3 (ser 10) abundance and distribution. Cells were transfected with FAK-siRNA and incubated for 48 h before usage in the coculture setup for a further 4, 7, or 10 days. Shown in (**A**) are representative IIF images of cryofixed sections of FAK-siRNA-treated versus nontreated cocultures, stained with an anti-YAP antibody. Likewise, epithelia were stained with an anti-pHH3 (ser 10) antibody after the indicated periods of time in (**C**). The bar graph in (**B**) shows the SQFI by measuring the relative integrated fluorescence density of the YAP fluorescence signal. In (**D**), quantification of the pHH3 (ser 10) fluorescence signal was performed. Both bar graphs compare the FAK-siRNA-inhibited cocultures with the noninhibited controls. (*n* = 3 for all experiments, values are depicted as mean ± SEM; * = *p* < 0.05. Scale bars represent 100 µm. NC = negative control; D = day).

As both the expression and spatial distribution of vinculin and ROCK-II are affected upon FAK knockdown, it was conceivable to assume that downstream mechanobiological effectors, such as YAP, would also respond to the intervention. This idea is supported by the notion that ROCK-II influences the stability and subcellular localization of YAP [74]. Thus, this transcriptional coactivator was investigated next. SQFI demonstrated roughly comparable amounts of YAP on day 4 and day 7, regardless of whether the gingival epithelial equivalents were treated with FAK-siRNA or not (Figure 8A: NCsi, D4 and D7, and Figure 7B: FAKsi, D4 and D7; Figure 8B). On day 10, a significantly lower YAP content was measurable within the control cultures, whereas the YAP protein level was maintained in FAK-siRNA-treated epithelia (Figure 8B, D10 and Figure 7A, D10: NCsi versus FAKsi). Of interest, YAP was predominantly localized in the superficial cell layers in the siRNA-treated epithelial equivalents. As YAP function depends on its subcellular localization, it would have been interesting to see whether the protein is localized cytoplasmically or within the nucleus. Unfortunately, it was technically impossible to separate the respective cell layers and to perform subfractionation of the cell lysates to distinguish between nuclear and cytoplasmic localization for each epithelial compartment. Therefore, YAP localization could only be analyzed visually from the fluorescence microscopy images. As regions of an unequivocal magenta fluorescence merger (overlapping of the red YAP fluorescence and the blue nuclear DAPI staining) were scarce, we assume that YAP is mainly localized cytoplasmically, which does, however, not exclude relevant amounts of nuclear YAP.

To obtain indirect information about YAP nuclear localization and function and the potential influence of the increase in pROCK-II^Ser1366^ in treated samples, pHH3^Ser10^, which is indicative for cell proliferation, was analyzed next. YAP is a potent driver of cell proliferation and was shown to promote epithelial hyperplasia, which we described for the FAK-siRNA-treated samples [75]. Consequently, an increase in proliferating keratinocytes in the epithelial equivalents would indirectly support the idea of an increase in YAP nuclear activity. Indeed, significant differences in cell nucleus pHH3^Ser10^ fluorescence were detected by SQFI particularly at day 7 and day 10 in FAK-siRNA-treated epithelia (Figure 8C: NCsi, upper panel; FAKsi, lower panel and Figure 8D). Quantification of pHH3^Ser10^ positive cells was performed by SQFI and not by manually counting the relative number of positive cells, because we recognized inhomogeneous cluster regions with high proliferative activity. To avoid a systematic error by accidentally counting these regions, we performed SQFI as a quantification method that considers the whole epithelial cross section and therefore more correctly represents the actual number of dividing cells. Taken together, the data represent indirect evidence for sustained keratinocyte proliferation upon FAK-siRNA treatment, which therefore at least in part explains the morphological hyperplasia of the epithelial equivalents. 

## 3. Discussion

The non-receptor tyrosine kinase FAK is employed in many physiological and pathological processes ranging from cellular adhesion and epithelial morphogenesis to cell migration and EMT. Although FAK’s role has been intensively studied in various human tissues, the herein-presented results show, for the first time, how FAK knockdown influences in vitro oral epithelial morphogenesis. 

The first step was to choose an appropriate in vitro model system, which would be suitable to investigate the role of FAK within the gingival epithelium. In skin research as well as in oral biology, various 3D models of the respective tissues have been developed to investigate the biological functions of certain molecules or to study the interaction of different cell types or drug effects [28,33,41,76,77,78]. Apart from avoiding animal experiments (3R principles: reduction—replacement—refinement), such platforms also offer perspectives for future regenerative approaches [65,79]. 

Current models mainly depend on the coculture of different organotypic cells, such as epithelial (keratinocytes) and stromal cells (fibroblasts). The epithelial−mesenchymal interface either consists of collagenous matrices, epidermis-free donor tissue, or biomaterials [26,29,67]. Although the former offer a more in vivo-like biochemical environment, we chose the latter approach for two reasons: Firstly, such a biomaterial can be fabricated in a manner that fits the tissue-specific needs from a biophysical and biochemical point of view. This includes an appropriate material stiffness, which is enormously important in the context of FA-dependent and FAK-related mechanotransduction, as well as a nanotopography which enables cellular adhesion. Secondly, biocompatible biomaterials offer the perspective for translation of the principles to cell-regenerative therapies in clinical and dental medicine [80]. In previous studies, we showed that an innovative nonwoven biohybrid membrane, which had a Young’s modulus of 3.2 kPa, was suitable to support gingival epithelial formation in vitro [32,38]. By employing this membrane, we could reproducibly generate organotypic epithelial equivalents of the human gingiva, which exhibited proper epithelial stratification and keratinocyte morphology. In comparison with the human gingiva, our epithelial equivalents, however, exhibited fewer cell layers (10–15 layers herein vs. 20–30 layers in vivo) [28]. This might be explained by the relatively short culture period of 10 days or other factors related to the experimental procedure. This does not, however, profoundly impair the significance of the presented results since key features like the biomarker expression strongly resemble the in vivo situation (see below).

Next, the appropriate cell types for our experiments were chosen. We decided to use primary GFs, since they are easy to harvest and cultivate and have been repeatedly shown to support gingival epithelial morphogenesis in vitro [63]. Immortalization of GFs is possible in principle, especially with human telomerase reverse transcriptase hTERT, but it is interference-prone, and the cells can undergo senescence after only 30–40 passages (own observation) [78,81]. The GKs we used had been immortalized with the E6 and E7 genes of the human papilloma virus HPV16. This immortalized cell line has previously been shown to enable in vitro epithelial formation with a proper epithelial stratification and biomarker expression. Although the use of primary GKs would be desirable from a regenerative point of view, primary GKs rapidly but uncoordinatedly differentiate under cell culture conditions, therefore resulting in a limited life-span. Moreover, they only show pseudostratification under postconfluent culture conditions, which do not allow for comparisons between in vitro and in vivo situations (own observation) [41,82,83]. Based on the aforementioned limited life span of primary keratinocytes, reproducible results are therefore only achievable with the immortalized cell line. Noteworthy, immortalization represents only the first feasible step in cell transformation, but does not lead to tumorigenic cells. This has been shown for immortalized skin keratinocytes (HaCaT) [84] and for our GKs, employed in the present study [41].

In comparison with skin keratinocytes, GKs show a different biomarker profile when analyzed under in vivo or in vitro conditions (e.g., Keratins 6, 13, 17 are found in the gingiva but not in the epidermis; other keratins are expressed in different cell layers when comparing gingiva and epidermis; for review see [28]). In the literature Keratin 1, 2, 4, 5, 6, 10, 13, 14, 16, 17, and 19 as well as vimentin and involucrin expression have been studied so far in native gingiva and/or in 3D experimental models. K1, K19, involucrin, and vimentin, therefore, served as reference points to compare our model with a previously reported experimental system. The expression of K1 in the upper spinous layer and the granular layer of our control samples is in accordance with the expression pattern of this IF in native gingiva [28]. In vitro expression of K19 in organotypic gingival cocultures was reported to be detectable foremostly in upper cell layers [27], as was the case with our epithelial equivalents. As expected, involucrin expression is predominant in apical cell layers within our control epithelia. 

Vimentin expression is stronger in our experimental system than reported for native tissue, which is frequently observed when primary or immortalized keratinocytes are cultured in vitro, where the growth-controlling influence of the mesenchyme is missing. Therefore, we initially detected vimentin in comparable amounts within our epithelial equivalents, while, due to the presence of the mesenchymal fibroblasts, it gets more and more downregulated in control epithelia [39,85,86]. 

Altogether, these results prove the feasibility and comparability of our experimental setup. This point is especially important, since other research groups did not use synthetic biomaterials but collagen matrices or de-epidermized connective tissue as substrates for in vitro epithelial formation. Within our study, we could even enhance the available panel of characteristic spatiotemporal protein expression patterns in gingival epithelial equivalents by additionally analyzing FAK, filaggrin, pVinculin^Y1065^, pHH3^Ser10^, YAP, and pROCK-II^Ser1366^, which is important for further characterization of our own model system and the comparison with other models. As seen above, these data show that gingival epithelial morphogenesis cannot be simply deduced from studies with skin keratinocytes/models. 

Since siRNA treatment inhibits the translation of mRNA transcripts that encode for certain proteins, we chose this approach to reduce FAK expression in the epithelial equivalents. The optimal siRNA amount, which leads to an efficient/sufficient (>70%) FAK inhibition in preliminary experiments, was evaluated. FAK-siRNA was superior to the FAK Y15 inhibitor, which yielded increased levels of pFAK Y397 in epithelial equivalents comprising GKs. These paradoxical results may be either explained by the above-mentioned EGF-related signaling or by other bypass mechanisms of FAK activation [71,72]. The bypass mechanism theory appears plausible, since studies on cancer cells revealed a direct resistance mechanism to FAK-kinase inhibition by cell surface receptor tyrosine kinases, which can preserve pFAK Y397 activity [87]. The mechanism of sustained FAK activation and chemotherapy resistance in cancer cells through neighboring cells has also been described for mantle cell lymphoma cells, which interact with bone marrow stromal cells. In this specific case, the combination of the treatment regime with pharmacological FAK inhibition could experimentally overcome the drug resistance and reduce FAK activity. Thus, it seems plausible that different cell types show a specific response to the mode of FAK inactivation and that the amount of the final FAK activity strongly depends on the exact biological context [88]. It will be interesting to further study the differences of pharmacological and siRNA-based FAK inhibition in different tissues and situations of cellular interaction since FAK abrogation is a promising concept for future cancer therapies, whose molecular basis should be evaluated as precisely as possible [5].

On the histological level, RNAi-mediated FAK shutdown resulted in increased numbers of cell layers at day 10. Morphologically, this resembles epithelial hyperplasia. This epithelial thickening coincides with increased protein levels of pHH3^Ser10^ at day 7 and day 10 when compared to nontreated controls. Histone H3 p^Ser10^ activity has been shown to begin in the early G2 phase of the cell cycle and is fully established during metaphase of mitosis (for review see [89]), which means that its detectability is a reliable indicator of cell proliferation. In accordance with our findings, Cortes and coworkers have also described the coincidence of increased proliferation with infection-induced small intestinal epithelial hyperplasia in mice and rats [90]. 

Regarding proliferation, former observations published by Gilmore and Romer, showed that FAK displacement from FAs resulted in reduced proliferation [34]. Novel molecular findings have broadened this concept, suggesting a dual role of FAK in proliferation. Herein, it has, for example, been proposed that G0-phase-synchronized FAK-/- mouse embryo fibroblasts revealed high proliferative activity. This indicates that loss of FAK has abolished the adhesion-dependent proliferative control mechanisms of cells and thus also the inhibitory role of FAK in cell proliferation [91]. Moreover, the pro-proliferative effects of FAK depletion were also demonstrated in cardiac fibroblasts, where FAK deficiency yielded increased proliferation [92]. These findings support the notion that the persistently higher levels of pHH3^Ser10^ detected in our GK-derived epithelial equivalents may be a consequence of the loss of the inhibitory role of FAK in response to RNAi-related FAK shutdown. However, the exact molecular mechanism by which FAK controls core players of cellular proliferation remains so far unclear. 

With respect to biomarkers of early and terminal keratinocyte differentiation, K1, involucrin, and filaggrin, FAK knockdown led to very different expression profiles compared to controls. In the case of K1 and involucrin, significantly higher protein levels were already detected on day 4, and the same applied to filaggrin on day 10. These findings suggest early commitment of cells to differentiation as a response to FAK protein shutdown and thus lead to imbalances within the gingival epithelium. To confirm the involvement of FAK in the regulation of differentiation within the epithelial equivalents, we analyzed whether the increased protein amounts of K1, involucrin, and filaggrin at the respective points of time correlated with increased levels of ROCK-II. We hypothesized that ROCK-II might be involved in this process since ROCK-II activity has been demonstrated to be important for keratinocyte differentiation [62]. In fact, our analysis revealed sustained and significantly higher ROCK-II activity, as indicated by pROCK-II^Ser1366^ analysis in epithelial equivalents treated with FAK-siRNA [93]. This indirectly confirms the participation of the FAK protein in the regulation of differentiation, which is a cornerstone of epithelial tissue homeostasis. In this context, it is important to distinguish between the FAK total protein and the activated pFAK Y397 fraction, since depletion of pFAK Y397 via mutation has been shown to exhibit no consequences on skin epithelia, i.e., epidermal differentiation in mice [70]. This again underscores that pharmacological FAK inhibition and siRNA-based approaches can yield different results. Another interesting facet of ROCK, and this applies to ROCK-I as well as to ROCK-II, is that both proteins are also involved in the regulation of proliferation. This involvement results from studies on ROCK-depleted cells, which are phenotypically characterized by a cell cycle arrest [60]. This fact, in addition to the previously discussed RNAi-related loss of the proliferation-regulating function of FAK, may also support the finding of sustained proliferation in siRNA-treated epithelial equivalents. 

In addition to the dual function of ROCK in both proliferation and differentiation, there is increasing evidence that ROCK also participates in the activation of the transcriptional coactivator YAP, which, amongst other functions, is also involved in the regulation of proliferation. Both ROCK-I and ROCK-II have been shown to regulate YAP activity, since prevention of nuclear YAP presence by ROCK inhibition abolishes transcription of YAP target genes in the case of ROCK-I [59], and any nuclear abundance at all, in the case of ROCK-II [74]. Based on these results, it appears possible that the strong ROCK-II activity detected in FAK-siRNA-exposed epithelia is associated with significantly higher amounts of YAP at day 10. Although we did not specifically analyze the subcellular localization of YAP, it cannot be ruled out that the persistence of YAP is causally related to the higher presence of the proliferation marker pHH3^Ser10^ upon FAK knockdown. 

While activated nuclear YAP promotes proliferation, cytoplasmic YAP has been reported to be present within the epidermal skin epithelium in all cell layers except for the basal cell layer. Thus, YAP localization coincides with the fraction of keratinocytes undergoing differentiation [94]. FAK-siRNA treatment led to an increase in YAP abundance with a predominant apical distribution within the epithelial equivalents. This finding provides evidence that the YAP protein detected in our cultures includes both subcellular localizations of YAP, namely the differentiation-associated cytoplasmic one, but also the nuclear fraction related to proliferation. In the YAP/FAK context we could show in a previous publication that within mesenchymal stem cells the cytoplasmic trapping of YAP leads to a nearly complete loss in the expression of FAK [58]. Based on this YAP/FAK correlation, it is conceivable that the depletion of FAK, in our case by RNAi, can result in an increased cytoplasmic presence of YAP. This in turn would explain the observed dysbalanced expression of GK differentiation markers, namely K1, filaggrin, and involucrin, which indicated premature GK differentiation throughout the epithelial equivalents and epithelial hyperplasia. 

Support for the assumption that our GK-derived epithelia exhibit nuclear as well as cytoplasmic YAP also arises from studies on TEAD (TEA domain proteins) family transcription factors, which, after binding YAP, initiate transcription. It could be shown that the inhibition of TEAD leads to an extended expression of involucrin and differentiation-indicating K10 in epidermal epithelial equivalents, which, together with K1, form intermediate filaments in keratinocytes [95]. Therefore, the expansion of K1 and involucrin towards the basal cell layers in our system may result from cytoplasmic YAP, which is then no longer available for binding to TEAD in the cell nucleus. Moreover, the extensive intraepithelial abundance of YAP after FAK knockdown is also consistent with the spatial expression patterns of the terminal differentiation markers, involucrin and filaggrin. Here, filaggrin showed a markedly greater expansion toward the basal region of the epithelia of treated GKs at day 10. With respect to involucrin, which also shows increased extension to the basal cell layer upon FAK knockdown at day 10, it should be noted that involucrin expression in cultured keratinocytes coincided with cytoplasmic YAP [96].

Keratinocyte differentiation begins at the interface of basal cells and the first suprabasal cell layer within stratified epithelia. At the same time, a redistribution of vinculin from FAs into AJs takes place, as FAs are lost in the first suprabasal cell layer and AJs are strengthened. The actin-binding protein vinculin is present in AJs and binds to α- and/or β-catenin in the context of mechanical loading of the cells [97]. Moreover, AJs are mandatory for proper keratinocyte differentiation [98,99]. In addition, the presence of vinculin is required for the formation of tight junctions during the early differentiation process within epithelia [100]. To detect vinculin in its actin-binding state, we used an antibody specific for Y1065 phosphorylation, since pY1065, amongst others, is required for a conformational change of vinculin to make it permissive for actin binding [101]. In the present study, the expression patterns of K1, involucrin, and filaggrin are in accordance with the spatial distribution detected for Y1065 phosphorylated vinculin. In the context of the above-discussed vinculin properties, this supports the notion that in our epithelial equivalents vinculin may undergo a switch from FAs to AJs. The predominant localization within AJs may then contribute to the induction and progression of keratinocyte differentiation. Again, this indirectly shows that FAK serves as a negative regulator of GKs differentiation under physiological conditions and is therefore a key factor for proper epithelial homeostasis.

Here, the FAK-directed RNAi inhibits the expression of the FAK protein per se. Although not tested experimentally, it can be assumed that the omission of the FAK protein affects the regulation of the genes for vimentin and K19 in the epithelial equivalents on both the expression level and the time scale. This assumption is based on publications, which report that FAK can shuttle between the cytoplasm and the cell nucleus and thus intervene in the regulation of gene transcription by interacting with a variety of transcription factors [102]. In the case of intermediate filaments, such as K19 and vimentin, the shutdown of FAK leads to a loss of its regulatory role in gene transcription. FAK-/- cells have been shown to exhibit altered intermediate filament expression [21,22,23]. Regarding K19, it has been proven that its overexpression reduces FAK expression in breast cancer cells [103]. Inversely, this finding provides indirect evidence that the FAK abrogation in our study may be involved in the persistently higher K19 expression. Furthermore, K19 has been shown to be required for proliferation in breast cancer cells [104]. Like ROCK, as discussed before, this may be another possible explanation for the significantly higher presence of pHH3^Ser10^ in FAK-siRNA-treated epithelial equivalents at day 7 and day 10. 

In addition to its role in EMT [86], vimentin has been shown to participate in the stabilization of FAK-containing FAs [50]. The increased expression of vimentin in FAK-siRNA-treated epithelial equivalents may therefore be an adaptive or compensatory response to the RNAi-induced loss of the FAK protein. This is because in vitro cell experiments with vimentin-deficient cells have shown that vimentin in FAs is partly responsible for the expression and phosphorylation of FAK [50]. These experiments support the hypothesis that loss of FAK in our siRNA-treated cultures results in increased expression of vimentin, as an increase in vimentin abundance enhances the cell’s probability of succeeding in its search for the binding partner FAK. In the previously cited paper, Havel and colleagues were also able to show that FAK co-immunoprecipitates with vimentin, suggesting a physical interaction of vimentin with FAK. Therefore, we propose a hypothesis in which FAK and vimentin expression and their protein levels are tightly coupled due to their physical and functional interrelationship. This has, of course, tremendous consequences for epithelial homeostasis as well as GK differentiation, as large amounts of vimentin are usually characteristic of connective tissue cells or the malignant transformation of epithelial cells. Thus, FAK seems not only to participate in GK proliferation and differentiation, but also to sustain the keratinocyte phenotype once established. Apart from the above-discussed molecular findings, which shed light on the mechanistic role of FAK and its tight interrelationship with cytoskeletal regulators, the filamentous systems and mechanotransducers in regulating gingival epithelial proliferation and differentiation, our study clearly showed how an innovative biomaterial, herein an electrospun nanofiber-based gelatin/PCL-nonwoven mat with a defined stiffness of 3.2 kPa, can be combined with cell biological techniques for studying cellular signaling pathways. Such approaches overcome many limitations of classical 2D cell culture studies, where the evolutive, spatiotemporal processes of oral epithelial morphogenesis, epithelial architecture itself, and cell-to-cell interactions in three dimensions cannot be represented adequately. This proof-of-principle study, therefore, presents a new research platform on which oral epithelial morphogenesis can be studied in a more in vivo-like manner by simultaneously reducing the need for animal experiments. This is an indispensable precondition to identify cellular key players, which are both necessary and sufficient for proper gingival epithelial morphogenesis in vitro and in vivo, as well as to define possible targets for their pharmacologic or RNA-based manipulation, e.g., in the treatment of precancerous epithelial lesions such as leukoplakia. Thus, from such experiments, as exemplified by the knockdown of FAK, the cellular consequences of the manipulation of various signaling proteins can be tested in vitro and in vivo and the corresponding results can then be used to optimize future regenerative treatment strategies. 

Taken together, the new data of the present work underline the central mechanistic role of FAK in oral epithelial morphogenesis as well as keratinocyte proliferation and differentiation. The molecular and histological findings in this innovative siRNA- and biomaterial-based approach therefore offer great possibilities for future approaches in oral regenerative medicine. 

We can therefore summarize the following key points, which highlight the research progress of our study:

(i) In vitro gingival epithelial morphogenesis with proper spatiotemporal expression of important GK biomarkers in the respective epithelial layers is feasible and reproducible with the herein presented biomaterial-based approach.
(ii) The presented experimental platform can therefore be used for various animal-free applications in the study of signaling networks within the 3D environment of epithelia or even to screen for organ-specific drugs. As mentioned above, as the membrane is biocompatible, the concept can also be used in future translational approaches, where patient-derived cells can be seeded on the scaffold, cultivated/expanded in the laboratory, and then be transplanted into the patient for defect coverage in critical-size injuries of the gingiva.
(iii) FAK-siRNA treatment in this 3D organotypic coculture leads to efficient FAK shutdown, whereas Y15 inhibitor treatments lead to paradoxical findings most likely due to signaling from the underlying fibroblasts.
(iv) Inhibition of FAK protein synthesis results in hyperplastic epithelia, which are more proliferative when compared to the control samples as indicated by proteins such as YAP, ROCK-II^Ser1366^, and pHH3^Ser10^. Additionally, differentiation biomarkers such as involucrin and filaggrin are more abundant, and their expression is more pronounced in basal cell layers.
(v) FAK knockdown in the gingival epithelial equivalents leads to aberrant expression of vimentin and K19, which should be studied further in the context of EMT and carcinogenesis.
(vi) As a consequence of (iii) and (iv), FAK signaling should not be inhibited via siRNA in the context of gingival tissue engineering and oral regenerative medicine, since FAK is necessary for proper epithelial morphogenesis and biomarker expression. Contrary to many cancer cell models, siRNA-based FAK inhibition is probably not a useful strategy to prevent epithelial overgrowth and the dysbalanced expression of GKs differentiation markers, as can be concluded from our data. Based on our findings, the role of FAK inhibition for the treatment of premalignant oral lesions needs to be studied in more detail.

## 4. Materials and Methods

Cell Culture. HPV 16 E6/E7-immortalized human gingival keratinocytes (GKs) were used as characterized before [41]. GKs were cultured in keratinocyte growth medium (KGM-2, Promocell, Heidelberg, Germany) containing the necessary supplements (Keratinocyte Growth Medium 2 Supplement Mix, Promocell, Germany) and 100 μg/mL kanamycin (Sigma-Aldrich, St. Louis, MI, USA). The GK cell lines were used up to passage 50 for the experiments. Cultures of primary gingival fibroblasts (GFs) were generated by the explant technique as described before [39]. Tissue harvesting from patients was performed according to the Helsinki Declaration, and approved by the institutional ethics committee (Reference Number: 411/08 121010: Experimental studies on human tissue specimens of the periodontium and primary cultures derived from periodontal tissues) [39]. Routine cell culture of GFs was performed in Dulbecco’s Modified Eagle’s Medium (DMEM) (Life Technologies Ltd., Carlsbad, CA, USA), containing 10% fetal calf serum (FCS) (Biochrom, Berlin, Germany), 2 mM l-Alanyl-l-Glutamine (Thermo Fisher Scientific, Waltham, MA, USA) and 100 μg/mL kanamycin (Sigma-Aldrich, St. Louis, MI, USA). Passages 8–11 of primary GFs were used.

Both GKs and GFs were cultured in 95% humidified atmosphere with 5% CO_2_ at 37 °C. Cells were harvested at 80% confluence before use in experiments. siRNA treatment. FAK-siRNAs (Si PTK2 ID 103,649 and ID103698) and negative control siRNA (catalogue number 4390843, determining nonspecific effects) were purchased from Thermo Fisher Scientific, USA and used according to the manufacturer’s protocol. The company offers a guaranteed siRNA efficiency via a validated siRNA-design algorithm. When using “Silencer Select pre-designed siRNA” to the same target, the two siRNAs will silence the target mRNA with an efficiency of 70% or more (Figure S1, supplementary data). GAPDH siRNA (catalogue number AM4624) was used as positive control to optimize siRNA delivery conditions and to reconfirm high levels of delivery in each RNAi experiment. Lipofectamine RNAiMAX (Thermo Fisher Scientific, USA) was used as transfection reagent. In preliminary experiments, Western blot analysis (supplementary Figure S1) showed that a final amount of 50 pmol FAK-siRNA enabled an efficient suppression of FAK expression in GKs. Thus, the amount of 50 pmol siRNA, corresponding to 25 nM siRNA concentration within the experiments, was used for all subsequent experiments.

Y15 Inhibitor treatment. For the inhibition of FAK activity (autophosphorylation at Y397), the FAK inhibitor Y15 (Sigma-Aldrich, SML0837, USA) was used. The effective inhibitory concentration of Y15 was validated for GKs in monolayer cultures, before using it in the coculture setup. Specifically, 2.5 × 10^5^ GKs were seeded per well in six-well plates in 2 mL KGM-2 medium containing the necessary supplements, as described above. The cells were incubated at 37 °C in a CO_2_ incubator until they reached 60–80% confluence. Three different inhibitor concentrations (10 μM, 20 µΜ and 30 μM) were tested. Following the monolayer validation test, the Y15 concentration of 10 μM was used for coculture experiments.

Construction of Epithelial Equivalents on Nonwoven Biohybrid Membranes. An innovative nonwoven biohybrid membrane, consisting of gelatin and polycaprolactone (PCL), was used as substrate for oral epithelium formation in vitro. The nonwoven membrane with an elasticity of 3.2 kPa had been produced according to the method described by Jedrusik et al. [32]. For the construction of in vitro human epithelial equivalents, a coculture system of GKs and GFs was used. Firstly, 1 × 10^6^ GKs in 80 μL KGM-2 medium were seeded on the top side of a 1 cm × 1 cm membrane and incubated for 3 h at 37 °C. After adhesion of the keratinocytes, FAK-siRNA, and control siRNA transfection experiments were conducted as described above for 48 h. On the following day, 2 × 10^5^ GFs in 80 μL DMEM medium were seeded on the other side of the membranes. The subsequent day, the membranes were carefully turned around with the GKs facing the lid of the culture dish. The day of this procedure was counted as day 1 of the actual coculture experiments. For the coculture procedure, FAD culture medium (KGM-2 medium and DMEM/Ham’s F-12 medium (PAN-BIOTECH, Aidenbach, Germany) at a 3:1 ratio)) was used and refreshed every second day. The GKs were then continuously exposed to the air−liquid interface of the cell culture system. For each point of time (day 4, 7, and 10), three independent biological replicates were established and analyzed by indirect immunofluorescence (IIF), Western blot (WB), or polymerase chain reaction (PCR), respectively.

HE staining and cell layers counting. The nonwoven biohybrid membranes containing epithelial equivalents were embedded in tissue freezing medium (Leica Biosystems, Australia) and cryofixed in liquid nitrogen at each point of time (day 4, 7, and 10). The cryoblocks were cut in sections (10 μm) using a Leica CM 1850 cryomicrotome and fixed on microscope slides. For hematoxylin and eosin (HE) staining, sections were fully immersed in Mayer’s hemalum (Merck, Darmstadt, Germany) for 5 min, followed by thorough rinsing in tap water for several minutes. Sections were then stained with eosin (1% [*w*/*v*], Merck, Darmstadt, Germany) for 45 s, again followed by thorough rinsing with tap water. Differentiation of the staining was performed with 100% ethanol for 1 min. Sections were dried and then covered with coverslips and Fluoromount-G (SouthernBiotech, Birmingham, AL, USA) embedding medium. For quantification of the cell layers of epithelial equivalents in both HE stained and DAPI stained sections (see below), the sections were assessed under the microscope by counting nuclei along the apico-basal axis (perpendicular to the membrane) of the epithelial equivalents. For each sample, the number of nuclei in three different regions of the section were counted and the mean value for each sample was used for statistical analysis. Three technical replicates of three biological replicates for each time point were counted manually, respectively and analyzed statistically. 

Indirect immunofluorescence (IIF). For the detection of different proteins, the section slides were fixed with pure, cold methanol for 5 min and were then treated with blocking solution (10% goat serum and 5% bovine serum albumin (BSA) in phosphate buffered saline (PBS)) for 60 min at RT. The slides were subsequently incubated with primary antibodies overnight at 4 °C. The following primary antibodies were used: total FAK (Abcam, Cambridge, UK, ab76496, 1:100), Involucrin (Abcam, Cambridge, UK ab53112, 1:100), Keratin 1 (Abcam, Cambridge, UK ab93652, 1:100), Filaggrin (Abcam, Cambridge, UK, ab81468, 1:200), YAP1 (Santa Cruz Biotechnology, Dallas, TX, USA, sc-376830, 1:100), phospho-Histone H3 (Ser10 (pHH3^Ser10^); Cell Signaling Technology, Frankfurt, Germany, 9701S, 1:100), phospho-Vinculin (Tyr(Y)1065; Invitrogen, Waltham, MA, USA, 44–1078G, 1:250), phospho-ROCK-II (Ser1366 (ROCK-II^Ser1366^); Thermofisher, PA5-34895, 1:200), Keratin 19 (Santa Cruz Biotechnology, Dallas, TX, USA, sc-6278, 1:50), Vimentin (Abcam, Cambridge, UK, ab92547, 1:100). After washing five times with PBS, the fluorochrome-conjugated secondary antibody (either anti-mouse IgG or anti-rabbit IgG, Thermo Fisher, Waltham, MA, USA, 1:200) was applied for 60 min at room temperature (RT). Nuclei were stained with 4’,6-diamidino-2-phenylindole (DAPI; 300 nM, Sigma Aldrich, St. Louis, USA) for 10 min at RT. Fluorescence images were documented using a Keyence BZ-9000 fluorescence microscope (Keyence GmbH, Neu-Isenburg, Germany). For each biological replicate, equal magnification and exposure time between the experimental sample and the corresponding negative control have been used for documentation for each antibody, respectively. Nuclei staining exposure time has been varied individually for optimized image quality. The pictures provided in the manuscript depict representative staining signals for each point of time and antibody of at least three biological replicates.

Western blot (WB) analysis. To obtain whole cell GK extracts, the epithelial equivalents were washed three times with PBS and the uppermost layer of the membrane scaffold containing GKs was separated from the whole membrane. Then, keratinocytes were transferred into a reaction tube and lysed for 10 min on ice with 400 μL RIPA buffer (Sigma-Aldrich, USA) supplemented with cOmplete Mini Protease Inhibitor Cocktail (Roche Diagnostics, Mannheim, Germany) and PhosSTOP phosphatase inhibitor (Roche Diagnostics, Germany). During the incubation period, the reaction tubes were vortexed each minute, before they were centrifuged for 10 min at 10,000 rpm at 4 °C. The supernatant was collected as the total protein extract. Total protein concentrations were measured using the Pierce BCA Protein Assay Kit (Thermo Fisher, St, Louis, MI, USA). For electrophoresis applications, 10 μg of total protein were separated by SDS-PAGE on 4–15% Criterion TGX Stain-Free precast midi gels (Bio-Rad Laboratories, Munich, Germany). Subsequently, proteins were transferred to 0.2 µm low-fluorescence PVDF membranes (Bio-Rad Laboratories, Munich, Germany) using the Trans-Blot Turbo Transfer System (Bio-Rad Laboratories, Munich, Germany). After blocking with Tris-buffered saline (TBS; Bio-Rad Laboratories, Munich, Germany) containing 0.2% Tween (Sigma-Aldrich, St. Louis, USA) and 5% bovine serum albumin (BSA; Sigma-Aldrich, St. Louis, USA) for 2 h at room temperature (RT), the PVDF membranes were incubated with the indicated primary antibodies (diluted in TBS-Tween, containing 0.5% BSA) overnight at 4 °C. The following primary antibodies were used: total FAK (Abcam, Cambridge, UK, ab76496, 1:1000), phospho FAK Y397 (Abcam, Cambridge, UK, ab81298, 1:500), involucrin (Abcam, Cambridge, UK, ab53112, 1:5000) and GAPDH (Santa Cruz, Dallas, Texas, USA sc-365062, 1:5000). Subsequently, membranes were incubated with horseradish peroxidase-labeled secondary antibodies (1:5000; anti-rabbit or anti-mouse, Life technologies, Carlsbad, USA) for 1 h at RT. The proteins of interest were detected using the Clarity Western ECL Blotting Substrate (Bio-Rad Laboratories, Munich, Germany) and imaged with the chemiluminescence application of the ChemiDoc Touch imager. The protein bands were normalized to GAPDH with ImageLab software (version 5.2.1; Bio-Rad Laboratories, Munich, Germany). For reference, see densitometric raw data in Supplemental Figures S3 and S4.

RNA isolation and quantitative RT-PCR. The relative gene expression of the keratinocyte differentiation markers Involucrin (IVL, RefSeq# NM_005547, Qiagen, Hilden, Germany), Keratin 1 (KRT1, RefSeq# NM_006121, Qiagen, Hilden, Germany) and Filaggrin (FLG, RefSeq# NM_002016, Qiagen, Germany) were assessed by semiquantitative real-time (RT) PCR.

Total RNA was isolated from GK epithelial equivalents (separation of GKs from GFs was performed as described for the WB) using the RNeasy plus mini kit (Qiagen, Hilden, Germany) as recommended by the manufacturer. RNA concentration and integrity were measured by an automated electrophoresis system (Experion-System, Bio-Rad Laboratories, Munich, Germany). Subsequently, cDNA synthesis was performed with 100 ng of RNA using RevertAid First Strand cDNA Synthesis Kit (Thermo Fisher Scientific, St. Louis, MO, USA) according to the manufacturer’s protocol using a C1000 Thermal Cycler (Bio-Rad Laboratories, Munich, Germany). RT-PCR reactions were carried out with the CFX96-RT-PCR Detection System (Bio-Rad Laboratories, Munich, Germany) using RT2 SYBR Green qPCR Master Mix (Qiagen, Hilden, Germany), and cDNA equivalent to 10 ng of total RNA. The targeted gene transcript levels were normalized to the housekeeping genes actin beta (ACTB; RefSeq#NM_001101, Qiagen, Hilden, Germany) and glyceraldehyde-3-phosphate dehydrogenase (GAPDH; RefSeq#NM_002046, Qiagen, Hilden, Germany). Data were collected and analyzed using CFX96 Manager Software version 1.0 (Bio-Rad Laboratories, Munich, Germany). 

Semi-quantitative fluorescence imaging (SQFI). SQFI is an established quantification method to calculate the relative protein expression of a protein of interest [82,105,106,107]. In brief, fluorescence photographs with a normalized exposure time and illumination settings were compared between different experimental groups. The channel corresponding to each specific antibody signal was imported into the image analysis software ImageJ. For analysis, in each image at least 3 different areas of epithelial morphology were circled as a region of interest (ROI). For each antibody, at least 3 section slides were likewise examined. The average intensity value was calculated and corrected for the background signal to yield the relative integrated density value. 

Statistical analysis. All experiments were performed in at least three biological replicates. All data are shown as mean ± standard error (SEM). For the statistical analysis, the GraphPad Prism software (GraphPad Company, San Diego, CA, USA) was used. Differences between each point of time were analyzed using Student’s t-test and were considered significant if *p* < 0.05.

## 5. Conclusions

This is the first study which describes the morphological and molecular consequences of siRNA-induced FAK shutdown in gingival epithelial equivalents established on an innovative biomaterial. The presented data not only shed light on the biological function of FAK, but also offer possibilities for prospective biomaterial-based oral epithelial regeneration. The FAK-shutdown-related consequences underscore the central mechanistic role of FAK in oral epithelial morphogenesis and the regulation of tissue homeostasis. Its tight interrelationship with key keratinocyte differentiation markers as well as core regulators of cellular adhesion and differentiation qualify FAK as a master regulator in gingival epithelial biology. These new insights are a first step in translating basic FAK research into future regenerative clinical approaches in oral biology and in a broader sense allow for a deeper understanding of the consequences of gene-knockdown-related effects on epithelial morphogenesis.

## Supplementary Materials

The following are available online at https://www.mdpi.com/article/10.3390/ijms22189774/s1.
