# Supplementary material for: FAK Shutdown: Consequences on Epithelial Morphogenesis and Biomarker Expression Involving an Innovative Biomaterial for Tissue Regeneration
Source: Int J Mol Sci. 2021 Sep 10;22(18):9774. doi: 10.3390/ijms22189774 (PMC8470904; doi:10.3390/ijms22189774)
Supplement: Supplementary file 1 [file ijms-22-09774-s001.zip › ijms-1347654-supplementary.pdf]

Supplementary material:

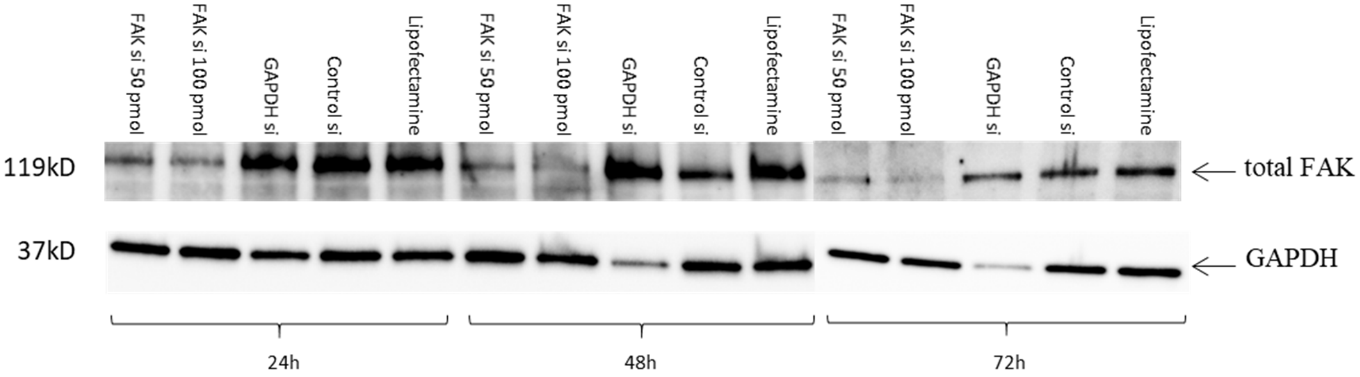

Figure S1: siRNA mediated FAK inhibition was determined in preliminary GKs monolayer experiments. It could be shown that application of FAK siRNA in different amounts (50 pmol = 25 nM or 100 pmol = 50 nM) led to an efficient FAK shutdown in GKs at all periods of time under investigation (24h, 48h, 72h). Lipofectamine, GAPDH siRNA, and a control siRNA served as negative controls. A representative Western blot from three biological replicates is shown.

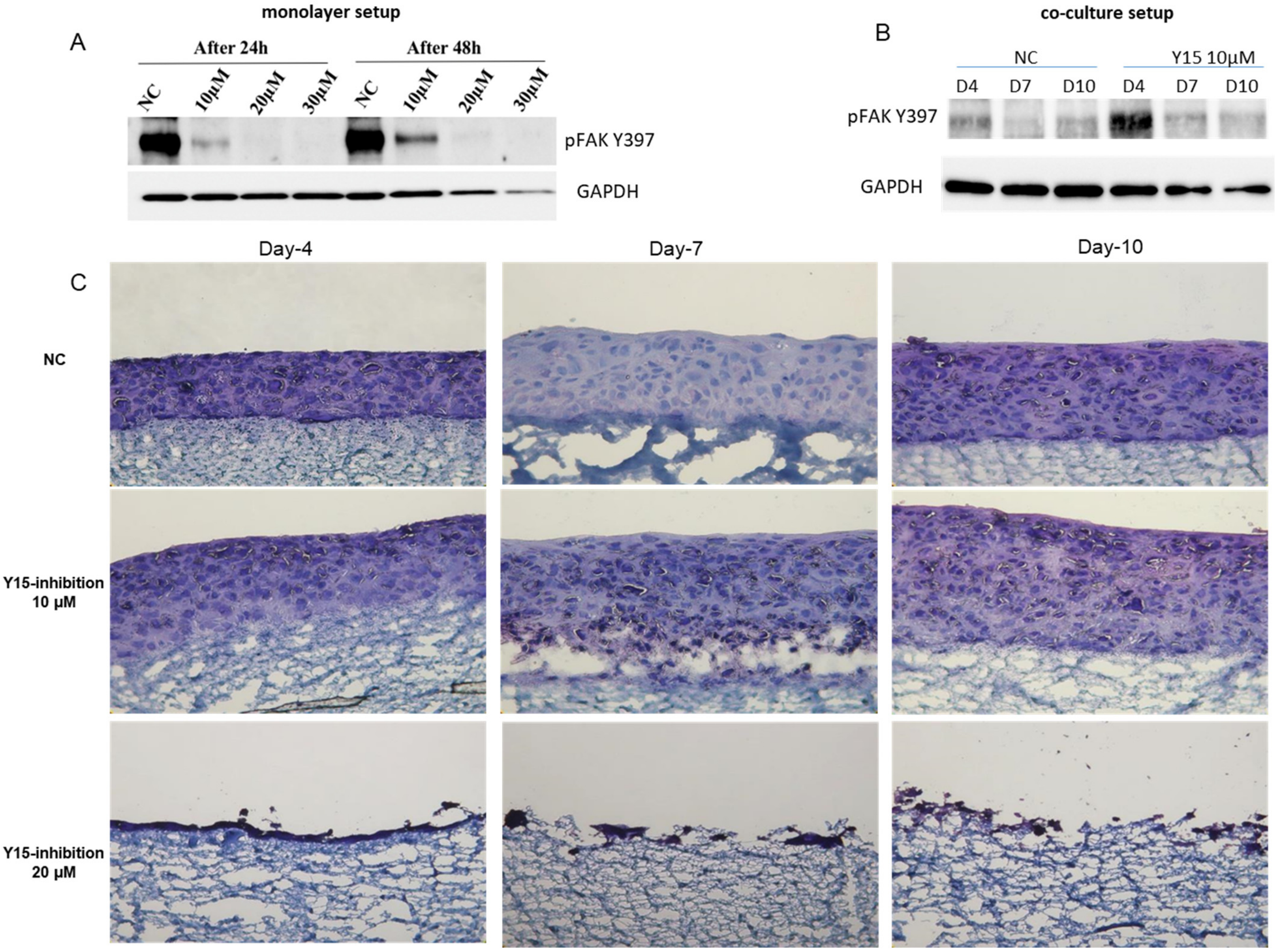

Figure S2: Y15-mediated inhibition of FAK in GKs-based mono- and co-cultures and the influence on pFAKY397 expression on epithelial morphology. Shown in **(A)** is a representative Western blot of pFAKY397 in Y15-

inhibited monolayer keratinocyte cultures. Different Y15 concentrations were tested for their efficiency to inhibit autophosphorylation of FAK at Y397 24h and 48h after inhibitor application. **(B)** GKs and GFs co-cultures were also treated with Y15 (10 μM) and analyzed for their pFAK Y397 protein abundance after 4, 7, and 10 days of inhibitor application. Untreated keratinocytes served as negative controls in **(A)** and **(B)** and GAPDH was used as a loading control. **(C)** shows cryo-fixed and HE stained sections of Y15-treated (concentrations as indicated) co-cultures of GKs and GFs versus non-treated co-cultures at the indicated points of time. NC = negative control; D = day; three biological replicates of all experiments were conducted.

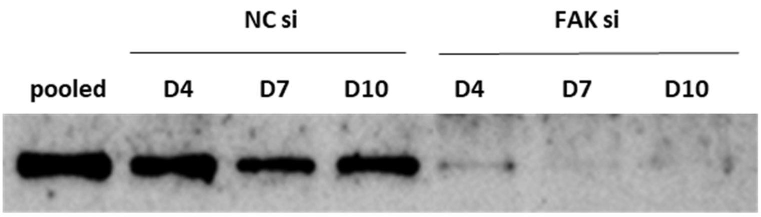

raw densitometric data

total FAK normalization							
Channel	Lane No.	Adj. Total Band Vol. (Int)	Total Band Vol. (Int)	Adj. Total Lane Vol. (Int)	Total Lane Vol. (Int)	Bkgd. Vol. (Int)	Norm. Factor
Chemiluminescence	1	28,914,533	18,245,823	77,018,459	148,157,662	80,112,942	1
Chemiluminescence	2	15,132,635	20,623,176	47,019,399	99,498,756	52,479,357	1.009480830
Chemiluminescence	3	7,017,549	10,246,614	39,007,010	88,160,555	49,153,545	1.076525913
Chemiluminescence	4	10,003,021	13,562,906	44,816,828	92,014,013	47,197,185	1.315364798
Chemiluminescence	5	2,044,420	3,986,500	46,684,533	93,637,173	46,952,640	1.031565012
Chemiluminescence	6	580,720	1,273,300	41,038,697	88,480,427	47,441,730	1.447120873
Chemiluminescence	7	599,522	1,347,794	37,565,444	88,822,076	51,256,632	0.952222302

normalized densitometric data

	total FAK	
	Adj. Total Band Vol. (Int)	norm band vol
pooled	28,914,533	100
NC si_D4	15,132,635	421.78845
NC si_D7	7,017,549	195.598527
NC si_D10	10,003,021	278.811901
FAK si_D4	2,044,420	56.9836479
FAK si_D7	580,720	16.1862748
FAK si_D10	599,522	16.7103387

Figure S3: siRNA-mediated inhibition of FAK in GKs-based co-cultures. Cells were transfected with FAK-siRNA and incubated for 48h before usage in the co-culture setup for further 4, 7, or 10 days. Shown is a representative western blot result of FAKsi-RNA treated cell extracts versus control cell extracts. The densitometric raw data were obtained using the chemiluminescence application of the ChemiDoc Touch imager. The protein bands were normalized to GAPDH with ImageLab software (version 5.2.1; Bio-Rad Laboratories, USA).

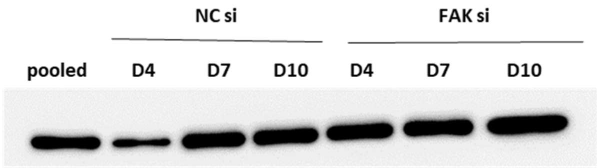

raw densitometric data

alisa 2021-05-05_14h53m32s	IVL						
Channel	Lane No.	Adj. Total Band Vol. (Int)	Total Band Vol. (Int)	Adj. Total Lane Vol. (Int)	Total Lane Vol. (Int)	Bkgd. Vol. (Int)	Norm. Factor
Chemiluminescence	1	67,902,328	71,034,656	72,951,086	146,238,468	73,287,382	1
Chemiluminescence	2	4,598,751	23,207,886	25,753,002	96,856,842	71,103,840	1.70008792
Chemiluminescence	3	62,594,937	91,536,723	93,412,227	164,570,925	71,158,698	1.37543374
Chemiluminescence	4	78,150,560	88,128,240	90,873,720	160,337,160	69,463,440	1.203817636
Chemiluminescence	5	88,991,115	92,507,931	94,672,854	165,811,134	71,138,280	1.07389986
Chemiluminescence	6	98,589,120	102,478,040	104,404,616	183,161,400	78,756,784	0.991056718
Chemiluminescence	7	130,946,260	136,047,916	37,644,664	109,272,768	71,628,104	0.970109071

normalized densitometric data

	IVL	
	Adj. Total Band Vol. (Int)	norm band vol
gepooled	67,902,328	85.11975651
NC si_D4	4,598,751	50.29189249
NC si_D7	62,594,937	198.5605322
NC si_D10	78,150,560	188.6601004
FAK si_D4	88,991,115	226.3630835
FAK si_D7	98,589,120	221.3502395
FAK si_D10	130,946,260	268.8924796

Figure S4: siRNA-mediated inhibition of FAK in GKs-based co-cultures and the influence on involucrin (IVL) expression. Cells were transfected with FAK-siRNA and incubated for 48h before usage in the co-culture setup for further 4, 7, or 10 days. Shown is a representative western blot result of FAKsi-RNA treated cell extracts versus control cell extracts. The densitometric raw data were obtained using the chemiluminescence application of the ChemiDoc Touch imager. The protein bands were normalized to GAPDH with ImageLab software (version 5.2.1; Bio-Rad Laboratories, USA).
